# King's College Hospital

**Published:** 1893-04-22

**Authors:** 


### FESTIVAL DINNERS.

### KING'S COLLEGE HOSPITAL.

The festival dinner of this hospital was held at the Hotel Metropole on the 13th inst., the Bishop of London presiding. Amongst those present were Mr. Justice Kekewich, Alderman Sir Joseph Savory, M.P., the Hon. W. Smith, M.P., Major-General Sir R. Pollock, Sir Paget Bowan, and many other distinguished personages. The Bishop of London proposed the toast "Prosperity to King's College Hospital," and pointed out that the best argument in favour of the hospital lay in the great number of patients whose sufferings had been relieved. Next to making a man better morally, the highest service that could be rendered him was to make him healthier. His Lordship drew attention to the fact that as at King's College the cost of one patient was but a guinea a year, most people could afford to subscribe such a sum, which might restore one fellow creature to health. The dependable income of the institution was but £12,000, whilst the expenses amounted to £18,000. The president, therefore, appealed for increased support. Donations resulting from the dinner amounting to £2,000 were announced. Messrs. W. H. Smith and Sons contributed £250 towards this, the Bishop of London £50, and Sir Horace Davey the same sum. The annual report of the institution shows that the number of in-patients during the past year has been 2,212, an increase by 137 on those received during the previous year, and 23,613 out-patients, showing an increase of 2,836.

